# Whole genome sequencing suggests transmission of *Corynebacterium diphtheriae*-caused cutaneous diphtheria in two siblings, Germany, 2018

**DOI:** 10.2807/1560-7917.ES.2019.24.2.1800683

**Published:** 2019-01-10

**Authors:** Anja Berger, Alexandra Dangel, Tilmann Schober, Birgit Schmidbauer, Regina Konrad, Durdica Marosevic, Sören Schubert, Stefan Hörmansdorfer, Nikolaus Ackermann, Johannes Hübner, Andreas Sing

**Affiliations:** 1 National Consiliary Laboratory for Diphtheria, Oberschleißheim, Germany; 2 Bavarian Health and Food Safety Authority, Oberschleißheim, Germany; 3 These authors contributed equally to this paper; 4 Division of Pediatric Infectious Diseases, Hauner Children's Hospital, Ludwig-Maximilians-University Munich, Munich, Germany; 5 Department of Health and Environment, Munich, Germany; 6 Max von Pettenkofer-Institute, Ludwig-Maximilians-University Munich, Munich, Germany; 7 These authors contributed equally to this paper

**Keywords:** diphtheria, toxigenic, Corynebacterium diphtheriae, WGS, MLST, outbreak, typing

## Abstract

In September 2018, a child who had returned from Somalia to Germany presented with cutaneous diphtheria by toxigenic *Corynebacterium diphtheriae* biovar *mitis.* The child’s sibling had superinfected insect bites harbouring also toxigenic *C. diphtheriae*. Next generation sequencing (NGS) revealed the same strain in both patients suggesting very recent human-to-human transmission. Epidemiological and NGS data suggest that the two cutaneous diphtheria cases constitute the first outbreak by toxigenic *C. diphtheriae* in Germany since the 1980s.

## Case reports

In early September 2018, a previously healthy school-aged child under 10 years old from a German family of Somalian origin presented in our hospital in Germany with an initially non-healing burn wound. The wound had occurred 6 days earlier when spilling hot tea on the right thigh during a flight back from Somalia to Germany. The child and close family members had spent the prior 3 weeks in Somalia. Wound swabs initially only led to growth of *Streptococcus pyogenes*, but subsequent wound swabs starting 10 days later led to growth of a toxigenic, toxin-producing *Corynebacterium diphtheriae* biovar *mitis* strain (isolate: KL1235). Since the patient fulfilled both the German [1] and European Union [2] case definition for diphtheria, this prompted their immediate hospitalisation and isolation according to the German national guidelines [1]. The strain was identified by biochemical differentiation (API Coryne code 1010324) and matrix-assisted laser desorption/ionisation time-of-flight mass spectrometry (MALDI-TOF MS; MALDI Biotyper; Bruker Daltonics, Bremen, Germany) [3]. Antimicrobial drug susceptibility testing of the isolate was performed on Mueller–Hinton blood agar (supplemented with 5% sheep blood) by Etest after overnight incubation at 37 °C and in 5% CO_2_. Minimum inhibitory concentrations were determined according to Clinical and Laboratory Standards Institute (CLSI) [4] and European Committee on Antimicrobial Susceptibility Testing (EUCAST) guidelines [5]. The isolate was resistant against both penicillin G and erythromycin, but sensitive towards clindamycin and amoxicillin/clavulanic acid. Toxigenicity was verified in the German Consiliary Laboratory on Diphtheria, Oberschleißheim, by real-time PCR and a modified Elek test [6]. 

Public health measures including source tracing among household and other close contacts were taken according to German national guidelines [1]. This revealed that the case had a one-year-older sibling who concurrently had a skin infection. This child was affected by multiple superinfected insect bites on the leg, which were already present during the stay in Somalia. A swab taken from a leg wound also led to growth of a toxigenic, toxin producing *C. diphtheriae* biovar *mitis* strain (isolate: KL1242). The strain had the same API Coryne code and antimicrobial resistance profile as the one in the younger sibling’s isolate prompting the child’s immediate hospitalisation and isolation. In addition, *S. pyogenes* could be isolated from the patient’s wounds in high concentrations, and *Pseudomonas stutzeri*, *Pantoea* species and *Arcanobacterium haemolyticum* were present in low concentrations.

To compare both *C. diphtheriae* strains, next generation sequencing (NGS) was carried out with both isolates as described previously, using Illumina Nextera XT libraries and an Illumina MiSeq [7]. Sequences were uploaded to the National Center for Biotechnology Information (NCBI) sequence read archive (SRA) [8], under BioProject PRJNA513482. Multilocus sequence typing (MLST) based on seven housekeeping loci [9] and extracted from the NGS data yielded sequence type (ST) 586 not previously found in the respective database [10]. NGS-derived core genome (cg)MLST comprising 2,154 target loci (1,553 core genome loci and 601 accessory genome loci) revealed no differences between the two isolates confirming strain identity. The NGS-based allelic profiles of the two isolates were compared with three Somalian and eight additional East-African *C. diphtheriae* isolates from an outbreak among African refugees in 2015 with potential transmission before arrival in Europe [11], as well as to three German and seven isolates from patients with travel or migration history to or from different other countries. The comparison showed no significant connections to any of the other isolates (Figure). 

**Figure:**
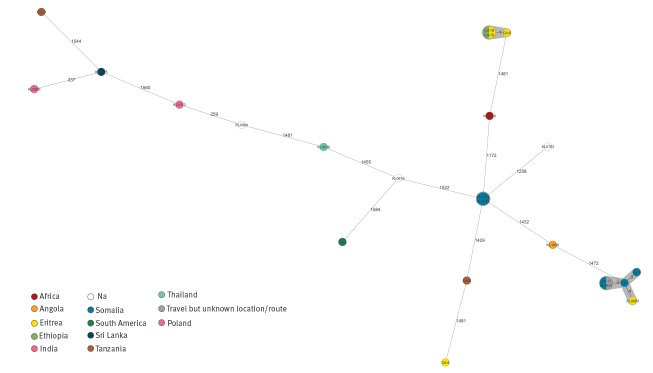
Minimum spanning tree based on next generation sequencing-derived allelic profiles^a^ of *Corynebacterium diphtheriae* strains, to investigate two isolates from siblings with cutaneous diphtheria who had travelled to Somalia, Germany, 2018 (n = 23 isolates)

Na: no travel information available.

Next generation sequencing-derived allelic profiles^a^ of two isolates (KL1235 and KL1242) recovered from siblings in Germany who had prior travelled to Somalia were compared with the profiles of 21 isolates recovered from persons with or without travel to/from Somalia and other countries. Isolates are colour-coded according to the country where the persons stayed prior to *Corynebacterium diphtheriae* infection diagnosis.

^a^ The allelic profiles were based on 1,553 core genome and 601 accessory genome target loci. Allelic differences between the strains are indicated and clusters of closely related isolates with maximum distance of five alleles are shaded in grey.

Both cases recovered quickly after antibiotic therapy with amoxicillin/clavulanic acid and wound cleansing. They were discharged home after they repeatedly tested negative for nasopharyngeal and wound *C. diphtheriae* carriage according to German infection management recommendations [1]. Both cases were fully immunised according to the German childhood vaccination recommendations including a booster vaccination at 4-6 years of age [12], as were all their close family members with the exception of one parent whose vaccinations were completed thereafter. All close household contacts, i.e. the family, tested negative for *C. diphtheriae* carriage, were offered antibiotic prophylaxis and were advised to self-monitor for development of diphtheria-like symptoms according to German recommendations. Since the older sibling reported to have demonstrated his superinfected insect bites to a large group of class mates, the local health department distributed leaflets on diphtheria among the school classes of both children. To date no secondary case has been detected.

## Discussion

Classical respiratory and cutaneous diphtheria are caused by diphtheria toxin (DT)-producing *C. diphtheriae*, *C. ulcerans*, and *C. pseudotuberculosis* that are spread by droplets or – especially in the case of cutaneous diphtheria – by direct contact. Due to the potential local or systemic spread of DT, classical diphtheria may give rise to severe respiratory symptoms as well as myocarditis and polyneuritis with a fatality rate between 5 to 30% [13]. In contrast, cutaneous diphtheria symptoms may be mild, unspecific and masked by co-infections but may be a source of secondary transmission and respiratory disease [13,14].

Neither the human source nor the geographical origin of the isolated *C. diphtheriae* strain reported here are known. Both siblings had returned from a three-week stay in Somalia where diphtheria might be endemic according to the last available diphtheria incidence data reported to the World Health Organization [15]: in 2012 Somalia ranked seventh of all countries worldwide with respect to the number of notified cases. Moreover, cutaneous diphtheria was identified among Somalian refugees to Europe in 2015 [11,16]. Cutaneous diphtheria cases have also been detected in Germany in recent years, albeit most, but not all of them, after travelling to endemic countries [11,16-18]. In the current report, the index case had received a burn wound on a flight from Somalia to Germany and presented at our hospital six days later, while back in Germany. Importantly, the swab which led to growth of *C. diphtheriae* was taken 16 days after the flight. There are several possible explanations for that: the child might have contracted the *C. diphtheriae* from their sibling who had reportedly acquired their subsequently superinfected insect bites when visiting Somalia. Supporting this hypothesis is the initial swab from the burn wound, which was negative for *C. diphtheriae*, in contrast to follow-up cultures during repeat visits in the surgical department, in which *C. diphtheriae* was identified. The other hypothesis would be that the index case might have been already colonised with *C. diphtheriae* in Somalia on either his skin or nasopharyngeal region from where the burn wound might have become superinfected. However, epidemiologically it cannot be determined exactly when and where, either of the two boys contracted the *C. diphtheriae* outbreak strain nor who infected whom. The MLST-derived ST 586 has previously not been described and therefore a geographical allocation of the source is not possible. Comparing NGS data from German and three Somalian *C. diphtheriae* with isolates from other countries gives no indication for a larger outbreak or a potential infection source in Germany, nor connection to a previously identified outbreak among Somalian and other East African refugees [11].

While the source of the *C. diphtheriae* strain remains elusive, we were able to prove the identity of both isolates by NGS suggesting human-to-human transmission between the two siblings and defining an outbreak according to the German Infection Protection Act [19]. Since the index case’s symptoms of cutaneous diphtheria developed considerable time after their initial burn wound, the diphtheria outbreak obviously initiated within Germany. To our knowledge, this is the first diphtheria outbreak in Germany since the early 1980s when the last outbreak was described in Wuppertal using phage typing as molecular typing tool [20]. Interestingly, an NGS-based proof of strain identity between patients as in our outbreak has so far only been documented for two couples of respiratory diphtheria patients and two asymptomatic carriers during a diphtheria outbreak in South Africa [21]. After the 1980s, to our knowledge no secondary cases or carriers within Germany have been identified following either a respiratory or cutaneous diphtheria index case. In contrast, a cutaneous diphtheria patient from the United Kingdom with a travel history to Ghana was recently reported to have transmitted toxigenic *C. diphtheriae* to a close contact presenting with nasal diphtheria [22]. In conclusion, cutaneous diphtheria should not be forgotten and can present a possible source for secondary diphtheria cases, therefore prompting adequate hygienic precautions.
